# Rational Regulation of Surface Free Radicals on TiO_2_ Nanotube Arrays via Ag_2_O–AgBiO_3_ towards Enhanced Selective Photoelectrochemical Detection

**DOI:** 10.3390/nano10102002

**Published:** 2020-10-11

**Authors:** Yajun Pang, Hao Chen, Jin Yang, Bo Wang, Zhenyu Yang, Jun Lv, Zhenghui Pan, Guangqing Xu, Zhehong Shen, Yucheng Wu

**Affiliations:** 1 School of Engineering, Zhejiang A&F University, Hangzhou 311300, China; yjpang@zafu.edu.cn (Y.P.); m18770916094@163.com (J.Y.); zhehongshen@zafu.edu.cn (Z.S.); 2 School of Materials Science and Engineering, Hefei University of Technology, Hefei 230009, China; 2017170142@mail.hfut.edu.cn (B.W.); 2019110272@mail.hfut.edu.cn (Z.Y.); lvjun@hfut.edu.cn (J.L.); ycwu@hfut.edu.cn (Y.W.); 3 Department of Materials Science and Engineering, National University of Singapore, Singapore 117574, Singapore; msepz@nus.edu.sg

**Keywords:** TiO_2_ nanotube arrays, AgBiO_3_, precipitation, high selectivity, photoelectrochemical sensors, organics detection

## Abstract

Due to integrated advances in photoelectrochemical (PEC) functionalities for environment detection applications, one-dimensional (1D) TiO_2_ nanostructures provide a new strategy (PEC sensors) towards organics detection in wastewater. However, the unidealized selectivity to the oxidation of water and organics limits the PEC detection performance. Herein, we designed a ternary photoanode consisting of Ag_2_O–AgBiO_3_/TiO_2_ nanotube arrays (NTAs) to solve this issue by using a facile one-step precipitation reaction. High oxidation capacity for organics is achieved by regulating the surface free radicals properly through the heterostructure formed between the interface of TiO_2_ and AgBiO_3_. More importantly, as a trap for electron capture, Ag_2_O in this ternary system could not only further improve the separation efficiency of charge carriers, but also capture electrons transferred to the TiO_2_ conduction band, thus reducing the electrons transferred to the external circuit and the corresponding background photocurrent when detecting organics. As a result, the reconstructed TiO_2_ NTAs decrease their photocurrent response to water and enhance their response to organics, thus presenting lower oxidation activity to water and higher activity to organics, that is, highly selective oxidation characteristics. This work provides more insights into the impact of charge transfer and surface free radicals on developing promising and efficient PEC sensors for organics.

## 1. Introduction

Global concerns over water pollution and its impact on the environment are becoming increasingly prominent, which has become a bottleneck problem in the current rapid development of the world. Water pollution issues are not only related directly to human health, but also seriously affect both economic and social development. To this end, water research has been included as one of the most momentous topics in modern environmental issues. Among these many studies, effective determination of organic compounds in wastewater is of great significance for improving water quality and reducing environmental load [1,2,3]. Up to now, the traditional methods, such as the potassium dichromate oxidation method, the potassium permanganate oxidation method, etc., have been widely used. However, these methods still have some shortcomings, including unsatisfactory reflux time and sensitivity, limited detection limit, secondary pollution, and high cost, which can no longer meet the increasing environmental requirements.

One-dimensional (1D) hollow nanostructures show respectable performance as photoanode materials due to their excellent photochemical property. They can provide large inner and outer surface areas and offer a direct path for electron transmission, resulting in improved carrier transportation and light scattering ability [4,5,6,7,8,9]. A kind of advanced 1D morphology for high-efficient photoelectrochemical (PEC) reaction is the self-organized titanium dioxide nanotube arrays (TiO_2_ NTAs) produced by electrochemical anodization on a Ti substrate [10,11,12,13]. Previous works have demonstrated the use of TiO_2_ NTA-based photoanodes for organic pollutant degradation and renewable energy production [14,15,16,17,18], in which TiO_2_ NTAs exhibit considerable performance due to their large surface area, high chemical and mechanical stability, oriented electron transport, and tunable morphologies [19,20,21]. Recently, inspired by the above-mentioned concept, detection sensors based on the PEC characteristic of TiO_2_ NTAs for organics have been established [22,23,24] and are considered to be a potential method to replace and solve these issues in the traditional way. Nevertheless, in practical applications, the recombination of photogenerated charge pairs and the undesired selective oxidation severely limit the PEC detection of TiO_2_ NTAs [22,25], where a large amount of energy from absorbed photons is lost because of heat and inefficient decomposition, resulting in low efficiency determination performance. Several strategies including hydrogen treating, precious metals loading, and semiconductor modification have been developed to promote the charge separation of TiO_2_ NTAs [19,23]. However, the necessity to simultaneously enhance the selective activity is still an urgent need but challenging when building high-performance PEC detection sensors for organics in wastewater.

Pentavalent bismuthate-based materials possess a valence band composed of O 2p and Bi 6s hybrid orbitals of holes, in which the good dispersion of Bi 6s orbitals promotes the migration of photogenerated carriers. Moreover, the anti-bonding between anion and cation is more conducive to the formation and flow of holes, which is obviously different from the nature of the TiO_2_ valence band (only O 2p orbital) [26,27]. Hence, it is reasonable to believe that Bi-containing materials will significantly regulate the surface free radicals of TiO_2_, thereby changing the surface electrochemical reaction and the complicated PEC process. In this study, we introduce both Ag bismuthate (AgBiO_3_) and Ag_2_O nanoparticles as co-catalysts to TiO_2_ NTAs by a one-step precipitation method. Impressively, the as-prepared ternary system presents enhanced PEC detection performance when compared with pristine TiO_2_ NTAs. As far as the specific detection process is concerned, both reducing the background current generated by water oxidation and improving the current response to the oxidation of organics have greatly improved the selective activity of our PEC sensor. Moreover, on the one hand, the complex PEC mechanism based on the thus-obtained ternary system was thoroughly explored and clarified by analyzing the complex detection reaction. On the other hand, we also revealed the important role of surface electrochemical reactions and corresponding surface free radicals in PEC detection.

## 2. Materials and Methods

### 2.1. Material Fabrication

The highly ordered TiO_2_ NTAs were prepared by anodic oxidation, as previously reported [22]. In detail, the working electrode of the Ti sheet and the counter electrode of graphite were put together into an electrolytic cell at a distance of 2 cm. The Ti sheet was carried out in a glycol electrolyte containing 0.15 M NH_4_F and 5 vol% H_2_O at 60 V for 6 h. Then, the as-synthesized TiO_2_ NTAs supported on the Ti substrate were ultrasonically vibrated in glycol to remove the covered debris. Finally, the anatase TiO_2_ NTAs were obtained by annealing in a muffle furnace at 500 °C for 2 h.

**Figure 1:**
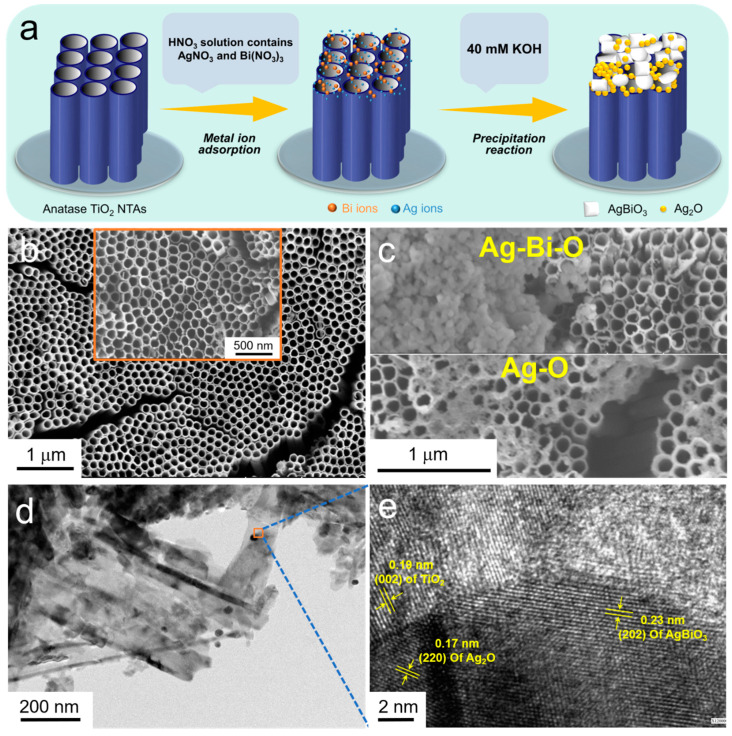
Schematic synthesis process and the corresponding morphology characterization of the co-modified titanium dioxide nanotube arrays (TiO_2_ NTAs). (**a**) Schematic representation of a two-step process to prepare co-modified TiO_2_ NTAs. (**b**) SEM image of pristine TiO_2_ NTAs. (**c**) SEM image, (**d**) TEM image, and (**e**) high-magnification TEM image of co-modified TiO_2_ NTAs.

The co-introduction of AgBiO_3_ and Ag_2_O nanoparticles was conducted by a precipitation method at room temperature. The as-synthesized TiO_2_ NTA samples were sequentially immersed in 1.3 M HNO_3_ solutions containing 40 mM AgNO_3_ and 20 mM Bi(NO_3_)_3_ (as Ag-Bi source) for 5 min, followed by the transfer to a 40 mM KOH solution to complete the precipitation reaction, resulting in the construction of ternary Ag_2_O–AgBiO_3_/TiO_2_ NTAs (see Figure 1a below). All reagents are purchased from Sinopharm Chemical Reagent Corp. (Shanghai, China) and used as received.

### 2.2. Characterizations

A scanning electron microscope (SEM, SU8020, Hitachi, Tokyo, Japan), a transmission electron microscope (TEM, JEM-2100F, Jeol, Akishima, Japan), an X-ray diffractometer (X′Pert PRO MPD, PANalytical, Etten Leur, The Netherlands, Cu Kα radiation), a LabRam HR Evolution instrument (Horiba Jobin Yvon, Palaiseau, France, excitation wavelength: 532 nm), an X-ray photoelectron spectrometer (ESCALAB250Xi, Thermo, Waltham, MA, USA, Al Kα monochromatic radiation), and a UV-3600 (Hitachi, Tokyo, Japan) spectrophotometer were used to study the properties of samples.

### 2.3. Photoelectrochemical Measurements

Photoelectrochemical (PEC) measurements were performed in a home-made flow injection device based on TiO_2_ NTAs measured in the thin-cell reactor, the details of which can be found in our previous literature [25]. The light source used in the experiment was a 365 nm UV LED source, whose diameter was 10 mm and whose usable power range was 0–1200 mW/cm^2^. The three electrodes used in the PEC test were the prepared sample (working electrode), Ag/AgCl (reference electrode), and Pt wire (auxiliary electrode). Glucose was used as target organics in this study. The photocurrent change and detection performance were measured by an amperometric method at a potential of 0.2 V in 0.05 M phosphate buffer solution (PBS, pH = 7), respectively. Specifically, PBS was pumped into the as-built reactor to obtain a stable current, that is, background photocurrent, and then a fixed concentration of glucose was injected to obtain a current response. The relationship between the increase in current and the concentration of organics was constructed by continuously injecting a certain concentration of glucose. The specific sequence of injection was twice 10 mM, twice 20 mM, once 40 mM, twice 50 mM, and then repeat 100 mM as much as possible. The impact of optical power on performance was studied by adjusting the power outputs from 4% to 8%.

## 3. Results

### 3.1. Microscopic Morphologies

Figure 1a presents the schematic diagram of the procedures for preparation of the co-modified TiO_2_ NTAs, the detailed preparation process of which is provided in Section 2.1, Material Fabrication. The nanostructure of the as-prepared samples in each step was investigated by using scanning electron microscopy (SEM). An orderly hollow 1D TiO_2_ NTA was first grown on a Ti substrate via the anodization method [28,29] (see Figure 1b). By comparison, two significantly different morphologies of reaction products can be observed on the surface of the NTAs after the reaction of one-step precipitation (Figure 1c). The treated samples still maintain an orderly and uniform nanostructure, which confirms that this strategy has no effect on the support structure. Figure S1 (Supplementary Materials) shows high-resolution SEM images and energy-dispersive X-ray (EDX) spectroscopy results of the co-modified TiO_2_ NTAs. It can be concluded that, in the area where the reaction product has a larger size, it is well distributed (Figure S1a,c), and the main elements are Ti, O, Bi, and Ag. In addition to the Ti and O elements from the TiO_2_ itself, two metal elements, Ag and Bi, have also appeared. Combining the existence of the valence state of the Bi element with a positive pentavalent value, the observed larger substances are bismuthate, while only the elements Ti, O, and Ag are detected in the area containing small particles. Moreover, it can be determined that the precipitate should be Ag_2_O due to the use of a KOH solution. The detailed nanostructures of the as-obtained products are further demonstrated by transmission electron microscopy (TEM) and high-magnification TEM. Large-sized AgBiO_3_ and Ag_2_O nanoparticles are distributed on the nanotube wall (Figure 1d). In addition, the high-magnification TEM image confirms the construction of our ternary nanoarrays. As shown in Figure 1e, the interplanar spacing of 0.19, 0.17, and 0.23 nm can be ascribed to the (002) plane of TiO_2_, the (220) plane of Ag_2_O, and the (202) plane of AgBiO_3_, respectively [30,31,32]. The interface of lattice fringes between TiO_2_, Ag_2_O, and AgBiO_3_ indicate the formation of a heterostructure, which promotes the electron transfer efficiency of nanoarrays in the PEC processes.

### 3.2. Crystalline Properties and Component Analysis

**Figure 2:**
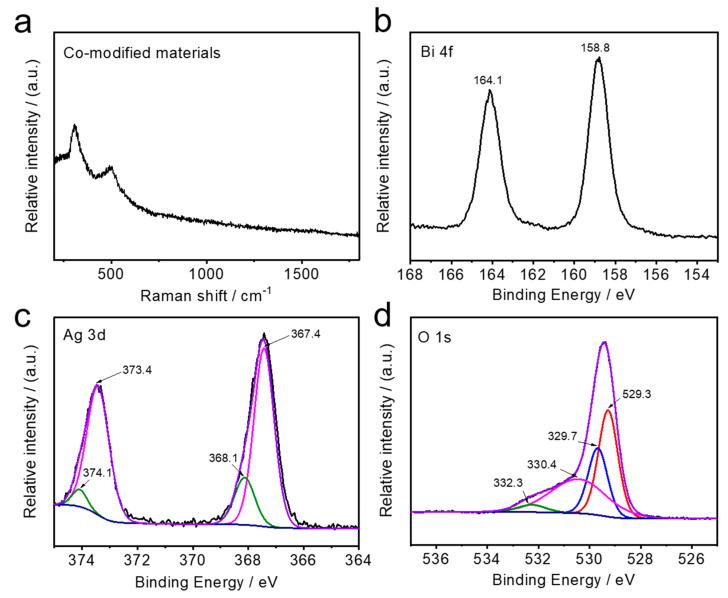
(**a**) Raman spectrum of the co-modified materials. XPS spectra of co-modified TiO_2_ NTAs: high resolution patterns of (**b**) Bi 4f, (**c**) Ag 3d, and (**d**) O 1s.

Prior to application, the products were annealed at 500 °C for 2 h in air to achieve high crystallization. The X-ray diffraction (XRD) patterns mainly reveal the anatase phase of the TiO_2_ material (PDF#21-1272) and the metallic phase of Ti (PDF#44-1294), which correspond to the TiO_2_ NTAs and the supported Ti sheet, respectively (Figure S2). In comparison with pristine TiO_2_ NTAs, no obvious other peaks can be observed in the reconstructed sample. This can be attributed to the low amount and crystallinity of the obtained AgBiO_3_ and Ag_2_O. Considering the preparation processes, we further used Raman spectra to record the reaction product between the two solutions (i.e., the co-modified materials) without nanotube support in Figure 2a, where the characteristic peaks can be attributed to Ag-O and Bi-O bonds, and is well consistent with AgBiO_3_ reported in the literature [31,33]. To better evaluate the obtained AgBiO_3_ and Ag_2_O grown on the TiO_2_ NTAs, we analyzed the chemical valence status and the corresponding surface elemental compositions of the co-modified TiO_2_ NTAs by X-ray photoelectron spectroscopy (XPS). The existence of the Ti, O, Bi, and Ag elements in the product is demonstrated by the broad-scan XPS survey patterns (Figure S3). The high-resolution XPS peaks of the main elements were also recorded to further investigate their detailed chemical states. The corresponding spectra of the Ag, Bi, and O elements are presented in Figure 2b–d. The peaks located at ~158.8 eV and ~164.1 eV are ascribed to Bi 4f_7/2_ and Bi 4f_5/2_, respectively (see Figure 2b). In general, the Bi element mainly shows three valence forms, namely zero, trivalent, and pentavalent. Here, by comparing the value of binding energy and previous studies, it can be concluded that the existing valence state of the Bi element synthesized by this precipitation method is a positive pentavalent ion (Bi^5+^ cations), which is consistent with the results observed in the above-mentioned SEM [26,34]. In the Ag 3d XPS spectrum shown in Figure 2c, the peaks at ~373.4 eV and ~367.4 eV can be attributed to Ag 3d_3/2_ and Ag 3d_5/2_. Furthermore, each peak can be analyzed into two peaks, where the peaks at 373.4 eV and 367.4 eV correspond to Ag ions in the AgBiO_3_ phase [34], while those at 374.1 eV and 368.1 eV are assigned to the Ag ion in the Ag_2_O phase [35,36], indicating that both AgBiO_3_ and Ag_2_O are formed from the precipitation reaction. Moreover, the XPS of the O 1s transition includes four distinct peaks (Figure 2d), that is, the peak at 529.7 eV is due to Ti-O (lattice oxygen), while the 530.4 eV and 532.3 eV peaks are in response to the Bi-O and C-O bonding structures, respectively. The peak located at 529.3 eV proves the existence of Ag-O in the final obtained product [37,38].

### 3.3. Surface Electrochemical Reactions and Related Free Radicals

In order to study the surface electrochemical reaction and free radical changes between TiO_2_ and the co-modified TiO_2_ NTAs, we sought more insight into all aspects involved in the PEC processes, including optical absorption, photogenerated charge separation and transfer, and the free radical trapping experiment. It has been well established that the optical absorption is the first step to activate the PEC processes for catalysts. Thus, a comparison of the optical absorption properties of the samples before and after being co-modified and investigated by UV–VIS diffuse reflectance spectra is displayed in Figure S4, which presents similar light absorption characteristics. Note that only UV light with a fixed wavelength of 365 nm was applied to perform the PEC measurement in this study, i.e., only the optical absorption of 365 nm position possesses the influence on the PEC processes for TiO_2_ and modified TiO_2_ NTAs. As marked in Figure S4, there is no obvious change in the optical absorption intensity of the co-precipitated sample when compared to the original sample. Therefore, it can be concluded that the optical absorption capability does not play a role in this system for the following PEC performance. We also measured the photoluminescence (PL) spectra to study the separation efficiency of the interface charge carrier in the obtained samples [39,40]. The emission peak of TiO_2_ and the co-modified TiO_2_ catalyst is observed at ~380 nm (Figure S5). The co-modified catalyst shows lower intensity compared with pristine TiO_2_ NTAs. It has been mentioned that the charge transport between different components in composite materials usually leads to a weakened PL emission band [40,41,42]. Moreover, the lower PL emission intensity demonstrates high efficiency separation and lower recombination of photogenerated charge pairs, which promotes PEC efficiency.

**Figure 3:**
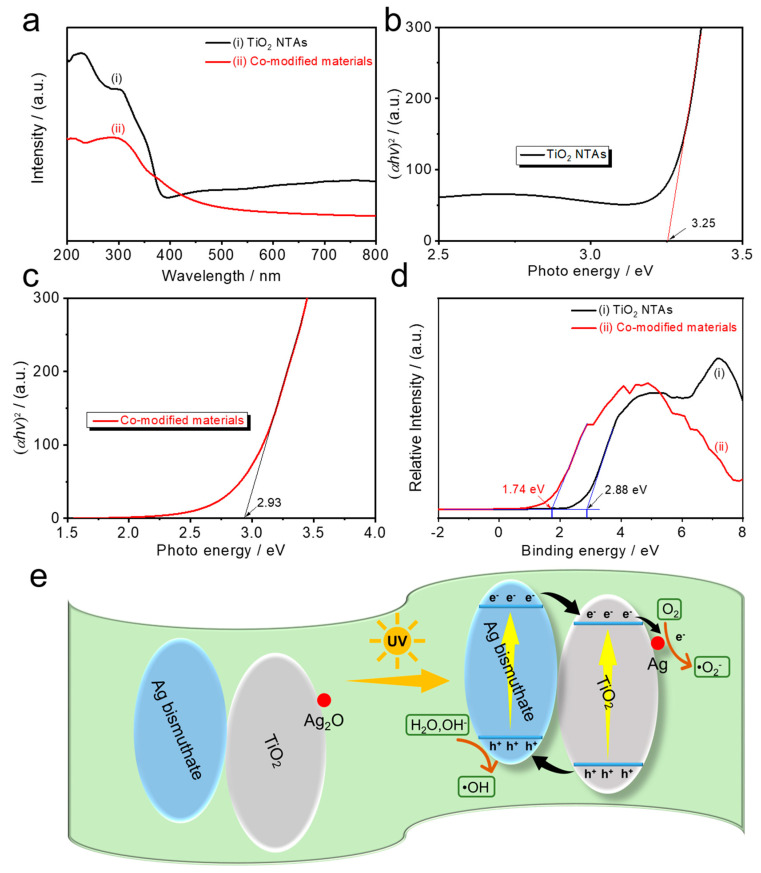
(**a**) Light absorption spectra, (**b**,**c**) transformed Kubelka–Munk function plotted against photon energy curves, and (**d**) XPS valance band edge of TiO_2_ and co-modified materials. (**e**) Schematic view for electron–hole separations and energy band matching of co-modified TiO_2_ NTAs under UV light irradiation.

In addition to optical properties and charge separation, elucidating the charge transfer path between TiO_2_ NTAs and co-modified materials is the core of exploring the changes in surface electrochemical and free radicals. We thus recorded light absorption spectra, curves of Kubelka–Munk function plotted against photon energy, and XPS valance band edge, respectively, to confirm their specific conduction band (CB) and valence band (VB) position. The above-mentioned Kubelka–Munk function plotted against photon energy curves was obtained by converting the individual UV–VIS diffuse reflectance of TiO_2_ and the co-modified materials [43], and it provided the specific band gap information (Figure 3b,c). Combined with the values of VB tested by the XPS valance band edges (Figure 3d), the specific values of CB and VB for both TiO_2_ and the co-modified materials were obtained. The migration between photogenerated charge carrier pairs was thus clarified. Eventually, the specific charge transfer paths in the ternary system were built, as illustrated in Figure 3e. Under illumination, the photogenerated charge pairs are active, and then the electrons transfer from AgBiO_3_ to TiO_2_; accordingly, the holes transfer from TiO_2_ to AgBiO_3_. In other words, the regulation of electrochemical surface reactions is achieved by introducing AgBiO_3_. More importantly, as the electron trapping, Ag_2_O could react with the electrons from the CB of TiO_2_ to restrict the recombination of photogenerated electron–hole pairs under UV light illumination [44]. This Ag_2_O-induced electron trapping also reduces the electrons transferred from the nanotube arrays to the external circuit through the Ti substrate, which could play a significant role in the regulation of background photocurrent in subsequent PEC detection applications.

**Figure 4:**
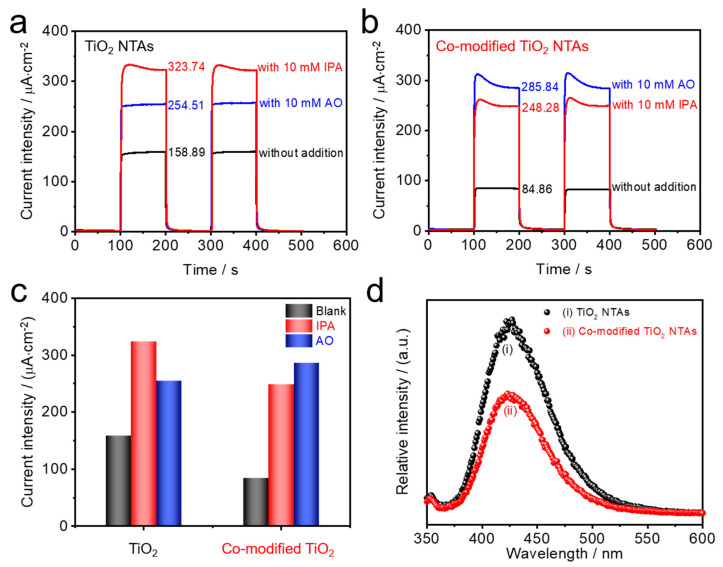
(**a**–**c**) Photocurrent of TiO_2_ and the co-modified TiO_2_ NTAs in PBS with capture agent added separately. (**d**) Photoluminescence pattern of terephthalic acid irradiated with ultraviolet light.

Changes in surface electrochemical reactions deeply affect the active surface free radicals of photocatalysts in the PEC reactions, especially the two reactions in the PEC detection process: (1) the direct organics oxidation by holes and (2) the indirect organics oxidation by hydroxyl radicals formed by holes [22,25,45,46]. Therefore, the corresponding changes in photocurrents of the as-prepared electrodes were studied by adding the corresponding radical trapping agents in PEC measurements (Figure 4a–c). Specifically, the holes and hydroxyl generated in the PEC process can be captured by ammonium oxalate (AO) and isopropanol (IPA), respectively. Different from hydroxyl is the main active radical for pristine TiO_2_ NTAs, where the holes become the main active radicals for the co-modified sample. Combined with the charge transfer path of co-modified TiO_2_ discussed above, we can see that the holes transferred to AgBiO_3_ have a lower activity to generate hydroxyl, due to the negative VB position [47], which explains the radical trapping agent experiment. Moreover, in order to extensively evaluate the intensity of hydroxyl, we measured the fluorescence intensity of 2-hydroxyterephthalic acid under 315 nm light excitation, which is the product of terephthalic acid after the reaction with hydroxyl from the catalysts’ surface (see Figure 4d) [48]. Obviously, compared with the original TiO_2_ NTAs, the recorded emission intensity is reduced, which confirms that the lower hydroxyl radical yield is obtained after the introduction of AgBiO_3_ and Ag_2_O. Such a phenomenon also proves the result of the trapping agent. Therefore, it can be demonstrated that the PEC detection of co-modified TiO_2_ NTAs prefers the direct oxidation of organics by holes, while the original TiO_2_ NTAs are indirectly oxidized by hydroxyl radicals.

### 3.4. Photoelectrochemical Detection Performance

**Figure 5:**
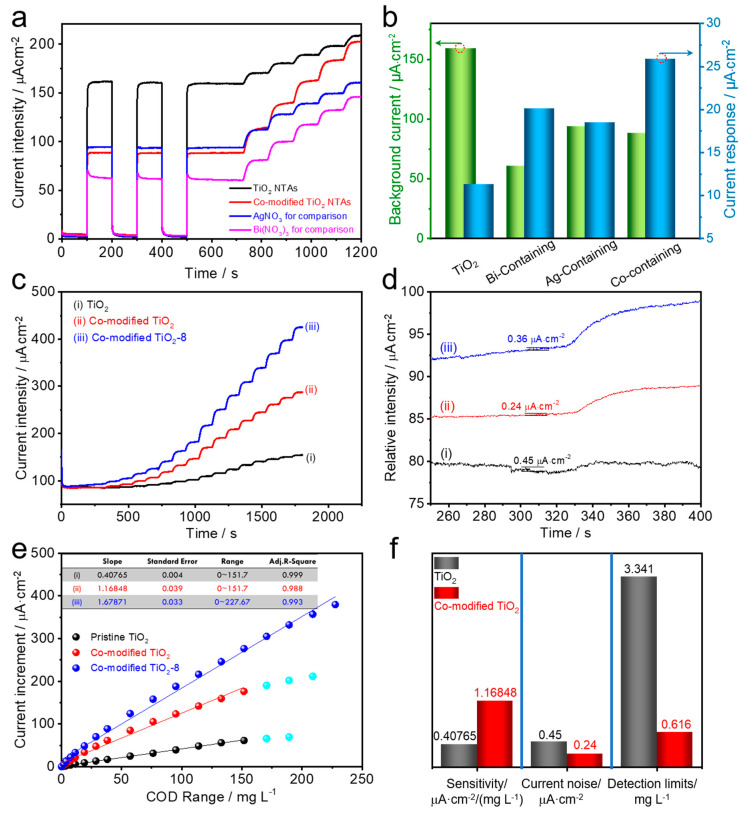
Photoelectrochemical (PEC) properties’ characterization. (**a**,**b**) Photocurrent and current response of TiO_2_ and the co-modified TiO_2_ NTAs. Amperometric response of the constructed PEC sensors to the continuous addition of glucose in PBS: (**c**) current intensity-time curve, (**d**) current noise obtained from the high-resolution curve of (**c**), (**e**) plots of current increments vs. chemical oxygen demand (COD) range, and (**f**) comparison of detection performances.

At this point, we can clearly conclude that the surface electrochemical reactions and related free radicals of the nanotube arrays were regulated due to the presence of silver bismuthate and silver oxide. Thus, we further investigated the photoelectrochemical detection performance towards organics of the as-constructed TiO_2_ NTAs. Specifically, in the case of PEC detection sensors towards organics in water, the photocatalysts simultaneously oxidize water and organics. This low selective oxidation causes a high background current (originating from the water oxidation), which reduces the efficiency and sensitivity of the assembled detection sensors. To investigate the selective oxidation ability of different samples, Figure 5a presents the background photocurrents to water and current responses of the 0.1 mM organics target continuously added, which was obtained on a self-made successive flow injection thin-cell PEC reactor system. Here, for comparison, we also conducted a comparative experiment involving single-metal and bimetallic precursors, that is, the samples was immersed in 1.3 M nitric acid solutions only containing 40 mM AgNO_3_ or 20 mM Bi(NO_3_)_3_ solution under the same conditions, respectively. We can observe that the pristine TiO_2_ NTAs possess a background photocurrent of 159.28 µA·cm^−2^ and a response of 11.33 µA·cm^−2^, whereas the background photocurrent of the co-modified TiO_2_ NTAs decreases to 88.49 µA·cm^−2^ and the current response increases to 25.90 µA·cm^−2^, both of which greatly benefit its application in PEC detection. As compared in Figure 5b, the Ag-containing/Bi-containing treated sample shows a photocurrent of 93.86 µA·cm^−2^/60.89 µA·cm^−2^ and a response of 18.50 µA·cm^−2^/20.14 µA·cm^−2^, which are better than those of pristine TiO_2_ NTAs but worse than those of co-modified TiO_2_ NTAs (see details in Table S1), which further demonstrates the superiority of bimetallic treatment. Overall, it can be concluded that the co-modified TiO_2_ possesses the best PEC performance in detection application among the above-mentioned samples. So far, it can also be concluded that the proper adjustment of surface free radicals and the oxidation reaction of organics (direct oxidation and indirect oxidation) discussed above can reduce the oxidation activity of the catalyst on water and at the same time enhance its oxidation activity on organics, which prove to be more advantageous in constructing a PEC detection sensor with high selectivity.

Based on the superior selective oxidation performance of the co-modified TiO_2_ NTAs, the PEC determination properties (range, sensitivity, and limit) were measured by an amperometric method with the successive addition of target [49]. By comparing the as-prepared co-modified TiO_2_ NTAs with the pristine TiO_2_ (curve (i) in Figure 5c) [22], we observed that they exhibit a rapid and superior response to the increase of chemical oxygen demand (COD) (converted from the concentration of organics). The current noise during the detection processes is also given by the amplified photocurrent curve (Figure 5d). It can be observed that the current noise is reduced by the precipitation treatment. The current increment vs. COD range was also calculated; as can be seen in Figure 5e, the linear part of the fitted curve provides the range and sensitivity of the constructed sensor. In addition, the detection limit (*dl*) of sensors, another important activity, is calculated by *dl* = 3*σ*/*m*, where *σ* is the noise of the recorded photocurrent and *m* is the slope of the linear part in Figure 5e [49]. The co-modified TiO_2_ NTAs present a much better detection performance (detection sensitivity, noise, and limit) when compared with the starting electrode, as seen in Figure 5f. Furthermore, by comparing this study with the reported literature, one can see that the co-modified TiO_2_ NTAs in this work exhibit competitive and comprehensive selective detection characteristics for organics (glucose), including sensitivity, range, and limit (see Tables S1 and S2 for details). Moreover, of particular interest, we found that the detection range could not be expanded. Therefore, an optical power that is twice the one previously used, namely 8%, was applied to study its impact (labeled as co-modified TiO_2_-8 in Figure 5e). It can be seen from the figure that, by changing the output power, the detection range can be easily adjusted, which also makes the materials show some flexibility in practical applications, such as adjusting to the suitable optical power according to different detection requirements.

## 4. Conclusions

In summary, the TiO_2_ NTAs co-modified with AgBiO_3_ and Ag_2_O were prepared by the one-step precipitation method, and their efficient application in a photoelectrochemical (PEC) sensor for the detection of organics was systematically investigated. In an as-built ternary system, as the formed charges were transferred between TiO_2_ and AgBiO_3_, the surface electrochemical reactions were appropriately adjusted. In addition, Ag_2_O played as an electron absorbent to further prevent electrons and holes from recombining. As a result, compared with pristine TiO_2_ NTAs, the rebuilt TiO_2_ NTAs exhibited lower oxidation activity to water and higher selectivity to organics, thus improving the PEC sensors’ efficiency when detecting organics in aqueous solution. The significant role of the active surface free radicals and the pathway for organics oxidation in boosting the comprehensive performance of PEC detection sensors was thoroughly explored and revealed.

## Supplementary Materials

The following are available online at https://www.mdpi.com/2079-4991/10/10/2002/s1, Figure S1: High-resolution SEM images and corresponding EDX spectroscopy results of the co-modified TiO_2_ NTAs; Figure S2: XRD patterns of TiO_2_ and co-modified TiO_2_ NTAs; Figure S3: Broad-scan XPS spectra of co-modified TiO_2_ NTAs; Figure S4. UV–Vis diffuse reflectance of TiO_2_ and co-modified TiO_2_ NTAs; Figure S5: PL emission spectra of TiO_2_ and co-modified TiO_2_ NTAs; Table S1. Comparison table of selectivity abilities for as-prepared samples; Table S2: List of reported parameters of TiO_2_-based PEC sensors for glucose detection.
