# Supplementary material for: Rational Regulation of Surface Free Radicals on TiO2 Nanotube Arrays via Ag2O–AgBiO3 towards Enhanced Selective Photoelectrochemical Detection
Source: Nanomaterials (Basel). 2020 Oct 11;10(10):2002. doi: 10.3390/nano10102002 (PMC7600186; doi:10.3390/nano10102002)
Supplement: Supplementary file 1 [file nanomaterials-10-02002-s001.pdf]

Supplementary Material

Rational Regulation of Surface Free Radicals on TiO_2 Nanotube Arrays Via Ag_2O–$AgBiO_3$ Towards Enhanced Selective Photoelectrochemical Detection

Yajun Pang [1,2], Hao Chen [1,*], Jin Yang [1], Bo Wang [2], Zhenyu Yang [2], Jun Lv [2], Zhenghui Pan [3], Guangqing Xu [2,*], Zhehong Shen [1] and Yucheng Wu [2]

1 School of Engineering, Zhejiang A&F University, Hangzhou 311300, China; yjpang@zafu.edu.cn (Y.P.); m18770916094@163.com (J.Y.); zhehongshen@zafu.edu.cn (Z.S.)
2 School of Materials Science and Engineering, Hefei University of Technology, Hefei 230009, China; 2017170142@mail.hfut.edu.cn (B.W.); 2019110272@mail.hfut.edu.cn (Z.Y.); lvjun@hfut.edu.cn (J.L.); ycwu@hfut.edu.cn (Y.W.)
3 Department of Materials Science and Engineering, National University of Singapore, 117574 Singapore, Singapore; msepz@nus.edu.sg
* Correspondence: haochen@zafu.edu.cn (H.C.); gqxu1979@hfut.edu.cn (G.X.)

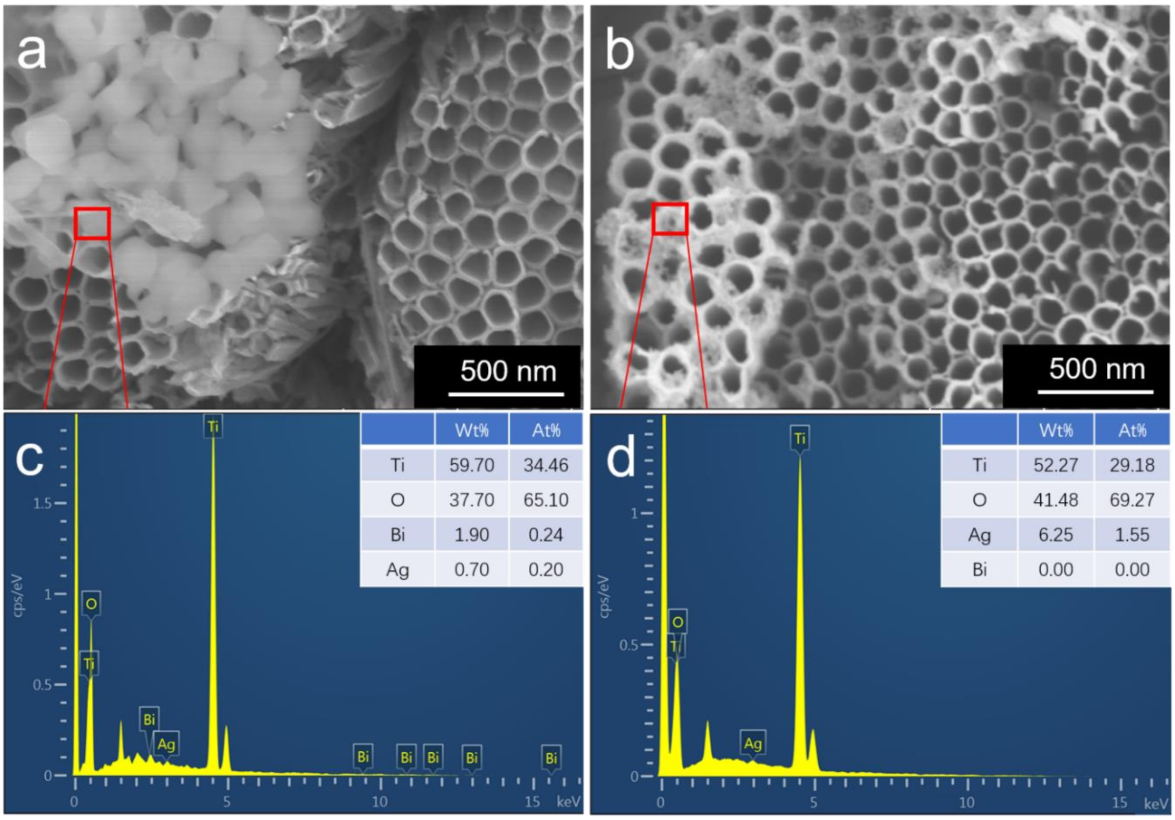

	Wt%	At%
Ti	59.70	34.46
O	37.70	65.10
Bi	1.90	0.24
Ag	0.70	0.20

	Wt%	At%
Ti	52.27	29.18
O	41.48	69.27
Ag	6.25	1.55
Bi	0.00	0.00

Figure S1. High-resolution SEM images and corresponding EDX spectroscopy results of the co-modified TiO_2 NTAs.

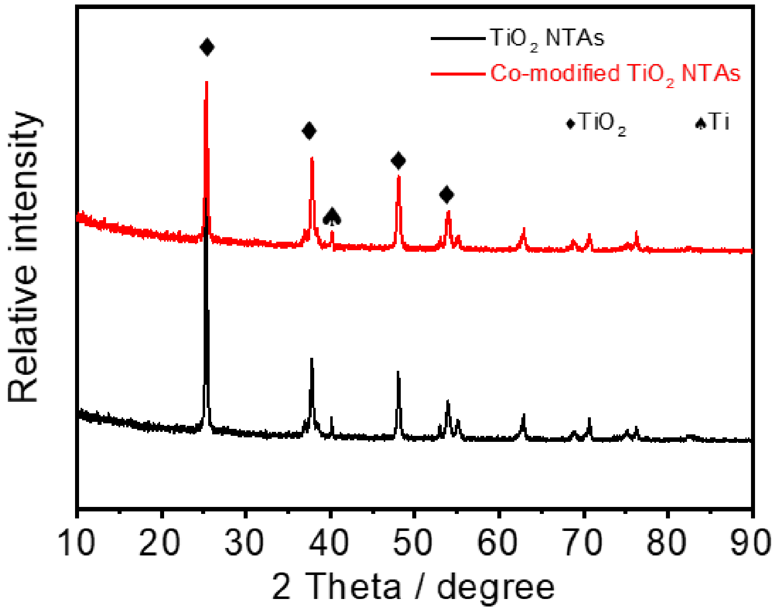

Figure S2. XRD patterns of TiO_2 and co-modified TiO_2 NTAs.

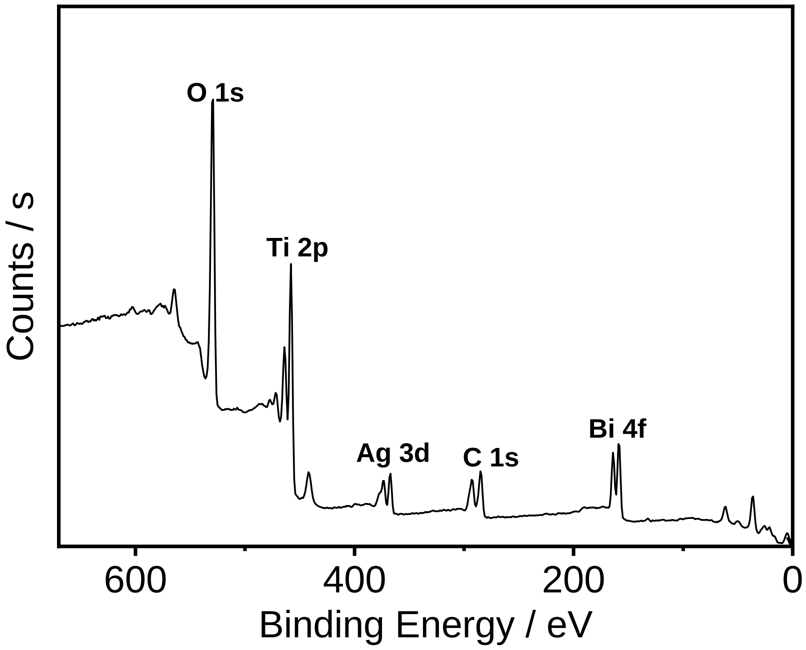

Figure S3. Broad-scan XPS spectra of co-modified TiO_2 NTAs.

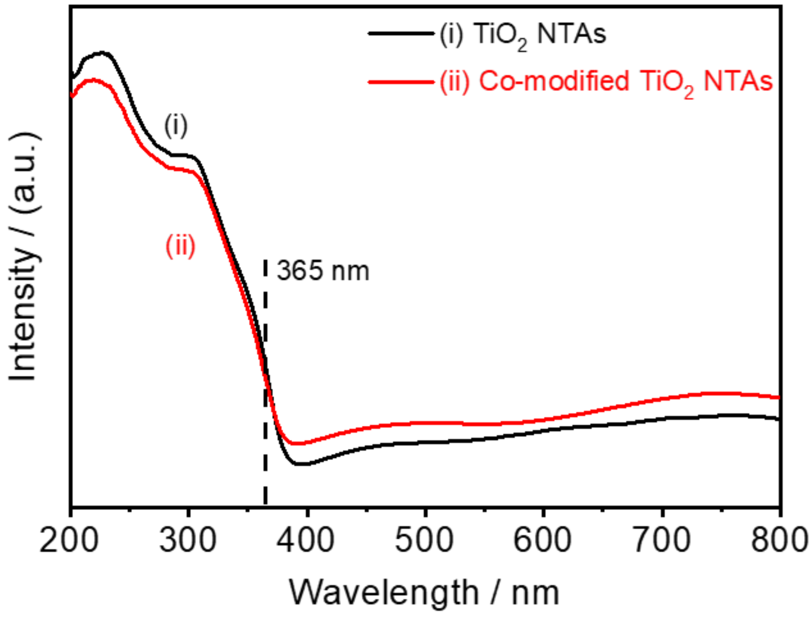

Figure S4. UV-Vis diffuse reflectance of TiO_2 and co-modified TiO_2 NTAs.

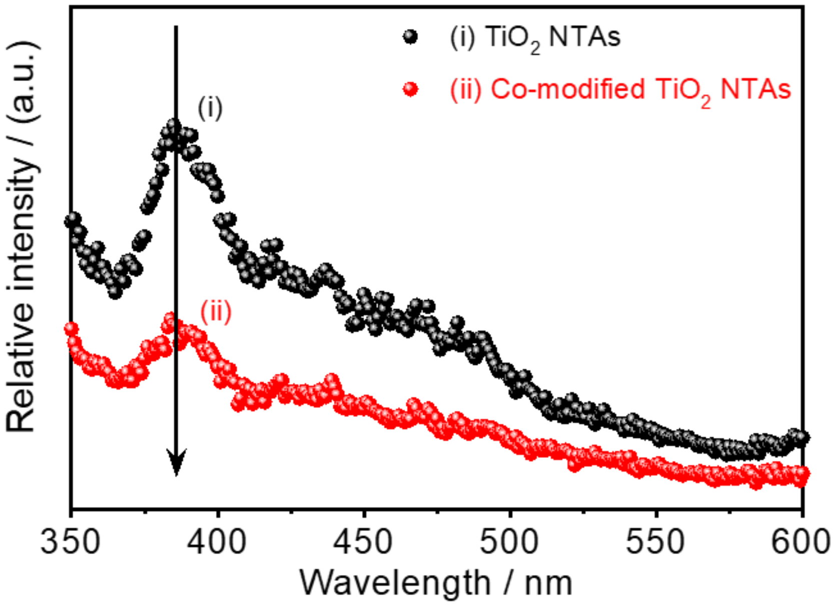

Figure S5. PL emission spectra of TiO_2 and co-modified TiO_2 NTAs.

Table 1. The comparison table of selectivity abilities for as-prepared samples.

	**Pristine TiO_2**	**Co-modified TiO_2**	**Ag-containing**	**Bi-containing**
Background photocurrent	159.28 $\mu A \cdot cm^{-2}$	88.49 $\mu A \cdot cm^{-2}$	93.86 $\mu A \cdot cm^{-2}$	60.89 $\mu A \cdot cm^{-2}$
Current response	11.33 $\mu A \cdot cm^{-2}$	25.90 $\mu A \cdot cm^{-2}$	18.50 $\mu A \cdot cm^{-2}$	20.14 $\mu A \cdot cm^{-2}$

Table S2. List of reported parameters of TiO_2-based PEC sensors for glucose detection. The symbol (#) indicates approximately the value extracted from the figure/data reported in each reference, which the author did not mention directly.

Sample	Wavelength	Sensitivity	Linear range	LOD	Refs.
Co-modified TiO_2	365	1.16848 μA·cm^{-2} / (mg L^{-1}) or 224.34816 μA·mM^{-1}·cm^{-2}	0-151.7 mg L^{-1} or 0-0.79 mM	0.6162 mg L^{-1} or 3.209 μM	This work
TiO_2	365	0.40765 μA·cm^{-2} / (mg L^{-1}) or 78.2688 μA·mM^{-1}·cm^{-2}	0-151.7 mg L^{-1} or 0-0.79 mM	3.341 mg L^{-1} or 17.401 μM	This work
Ag/TiO_2	365	194 μA·mM^{-1}·cm^{-2}	0-0.7 mM	0.53 μM	[1]
Bi/TiO_2	365	254.7 μA·mM^{-1}·cm^{-2}	0-0.5 mM	4.2 μM	[2]
Bi_2WO_6/TiO_2	365	196.1 μA·mM^{-1}·cm^{-2}	0-0.6 mM	3.31 μM	[3]
Pt/TiO_2	Xenon lamp	56 μA·mM^{-1}·cm^{-2}	0.1-4.5 mM	20 μM	[4]
Au/CuS/TiO_2	White light	#178 μA·mM^{-1}·cm^{-2}	0.1 - 3 μM	0.03 μM	[5]
Ti/TiO_2-rGO-Cu_2O	halogen lamp	0.87 μA·mM^{-1}·cm^{-2}	0.0007-20 mM	0.21 μM	[6]
TiO_2-MoS_2-GOx	Xe lamp	0.81 μA·mM^{-1}·cm^{-2}	0.1–10 mM	15 μM	[7]

References

1. Xu, G., Liu, H., Wang, J., Lv, J., Zheng, Z., & Wu, Y. (2014). Photoelectrochemical performances and potential applications of TiO_2 nanotube arrays modified with Ag and Pt nanoparticles. Electrochimica Acta, 121, 194-202.
2. Hu, J., Xu, G., Wang, J., Lv, J., Zhang, X., Zheng, Z., & Wu, Y. (2014). TiO_2 nanotube arrays modified with Bi nanoparticles for enhancing photoelectrochemical oxidation of organics. Journal of The Electrochemical Society, 161(9), H529.
3. Pang, Y., Xu, G., Zhang, X., Lv, J., Shi, K., Zhai, P., ... & Wu, Y. (2015). Photoelectrochemical properties and the detection mechanism of Bi_2WO_6 nanosheet modified TiO_2 nanotube arrays. Dalton Transactions, 44(40), 17784-17794.
4. Cai, J., Huang, J., Ge, M., Iocozzia, J., Lin, Z., Zhang, K. Q., & Lai, Y. (2017). Immobilization of Pt nanoparticles via rapid and reusable electropolymerization of dopamine on TiO_2 nanotube arrays for reversible SERS substrates and nonenzymatic glucose sensors. Small, 13(19), 1604240.
5. Wang, Y., Bai, L., Wang, Y., Qin, D., Shan, D., & Lu, X. (2018). Ternary nanocomposites of Au/CuS/TiO_2 for an ultrasensitive photoelectrochemical non-enzymatic glucose sensor. Analyst, 143(7), 1699-1704.
6. Çakıroğlu, B., & Özacar, M. (2019). Photoelectrochemical and Non-Enzymatic Glucose Sensor Based on Modified Fehling's Test by Using Ti/TiO_2 NTs-rGO-Cu_2O Electrode. Journal of The Electrochemical Society, 166(8), B728.
7. Liu, X., Huo, X., Liu, P., Tang, Y., Xu, J., Liu, X., & Zhou, Y. (2017). Assembly of MoS_2 nanosheet-TiO_2 nanorod heterostructure as sensor scaffold for photoelectrochemical biosensing. Electrochimica Acta, 242, 327-336.
